# External treatment of traditional Chinese medicine for functional dyspepsia in children: Protocol for a systematic review and network meta-analysis

**DOI:** 10.1097/MD.0000000000031597

**Published:** 2022-10-28

**Authors:** Junyu Cui, Jiaxin Wang, Ying Wang, Chuang Zhang, Guanyu Hu, Zhihong Wang

**Affiliations:** a Department of Acupuncture and Tuina, Changchun University of Chinese Medicine, Changchun, China; b Department of Endocrinology, The Second Affiliated Hospital of Changchun University of Chinese Medicine (Changchun Hospital of Chinese Medicine), Changchun, China; c Orthopedics and Traumatology Department of Traditional Chinese Medicine, The Third Affiliated Hospital, Southern Medical University, Guangzhou, China.

**Keywords:** children, functional dyspepsia, network meta-analysis, systematic review

## Abstract

Functional dyspepsia (FD) is a common digestive system disease in pediatrics, usually affects normal growth and increases health care expenditure. Many investigations have demonstrated that external treatment of traditional Chinese medicine (TCM) has certain advantages in the treatment of FD children, but the effectiveness, safety, and advantages of various methods have not been confirmed by high-quality meta-analyses. This study will conduct a systematic review and network meta-analysis to evaluate the differences and effectiveness in external treatments of TCM, in order to provide a reference for further clinical treatment for FD in children.

**Methods:**

Nine electronic databases, including PubMed, Medline, Embase, Web of Science, Cochrane Central Register of Controlled Trials, China National Knowledge Infrastructure, Chinese Biomedical Literature Database, Chinese Scientific Journal Database, Wan-Fang Database and one clinical trial register platforms: ClinicalTrials.gov (www.ClinicalTrials.gov/) will be searched using English and Chinese search strategies. All eligible studies are randomized controlled trials of TCM external treatment for FD in children, published on or before July 20, 2022. The screening process will be developed by 2 independent authors, and network meta-analysis will be performed with RevMan (V5.3) software.

**Results:**

This study will provide a high-quality synthesis to assess the effectiveness and safety on the external treatment of TCM for children with FD.

**Conclusion:**

The results of this study will provide evidence to judge whether the external treatment of TCM are effective interventions for children with FD.

**Ethics and dissemination:**

The results of this meta-analysis and meta-regression will be disseminated through publication in a peer-reviewed journal and presented at a relevant conference. The information used in the network meta-analysis does not contain individual patient data. Therefore, ethical approval was not required.

**PROSPERO registration number:**

CRD42022360429.

## 1. Introduction

Functional dyspepsia (FD) is a widely prevalent problem in pediatrics. Based on the predominant symptoms, FD can be subdivided into diagnostic subtypes of postprandial distress syndrome (predominant symptoms include postprandial fullness and early satiety) and epigastric pain syndrome (predominant symptoms include epigastric burning and epigastric pain), with the former subtype being more prevalent in Asia.^[1,2]^ In Western countries, the annual prevalence rate of adults is as high as 25 %, accounting for 2 % to 5 % of primary health care visits. Prevalence of 3% to 27% is described in children, depending upon community and school-based studies.^[3,4]^ Twelve percent to 16% of children referred to tertiary care clinics in the U.S. have dyspepsia.^[5,6]^ However, the underlying pathology of FD is not fully understood, it is considered to be related to upper gastrointestinal inflammation, gastrointestinal motility and sensory dysfunction, visceral hypersensitivity, Helicobacter pylori infection, gastrointestinal hormones, brain-gut axis.^[7–9]^ Western medicine can only relieve the symptoms of a few patients, and has obvious adverse reactions to children.^[10,11]^ Therefore, it is very important to select a safe, cost-effective treatment for the treatment of functional dyspepsia in children.

Nowadays, researchers are showing their interest in non-pharmaceutical therapies for FD in children, as there may have some side effects of conventional drugs’ using. A number of randomized controlled trials have shown that external treatment of traditional Chinese medicine (TCM) has certain advantages over conventional drugs in the treatment of FD in children.^[12]^ Therefore, this study will conduct a network meta-analysis to discuss the effectiveness, safety, and advantages of the external treatment of TCM, and compare the differences between them, to provide reference for further clinical treatment.

## 2. Methods and design

This study is based on the Preferred Reporting Items for Systematic Reviews and Meta-Analyses Protocols (PRISMA-P) guidelines^[13]^ and the corresponding checklist used. The Bayesian network meta-analysis for this study has been registered on the PROSPERO International Prospective Registry of Systematic Reviews (ID: CRD42022360429).

### 2.1. Inclusion criteria

#### 2.1.1. Types of studies.

Only randomized controlled trials on the external treatment of TCM for FD in children published on or before July 20, 2022 will be included in this study. All eligible studies languages will be limited to Chinese and English, but no limited to countries and publication status.

#### 2.1.2. Types of participants.

Patients under 18 years old diagnosed with FD without other comorbidities will be included in this study. There will be no restrictions on race, gender and disease course.

#### 2.1.3. Types of interventions.

The treatment group adopted the external treatments of TCM, including acupuncture, moxibustion, massage, acupoint application alone or in combination, while the control group adopted internationally recognized western medicine (such as domperidone), we have no restrictions on the administration method and course of treatment.

#### 2.1.4. Outcomes.

Results include effectiveness and safety. The primary outcome measure will be the extent of the clinical symptomatic remissions. Included at least one of the Gastrointestinal symptom rating scale and Leuven postprandial distress scale. The additional outcomes are the incidence of adverse events and the recurrence rate of 1 year after treatment.

### 2.2. Database search strategy

The search strategy will be based on the Cochrane handbook guidelines (5.1.0). We will search 9 electronic databases, including PubMed, Medline, Embase, Web of Science, Cochrane Central Register of Controlled Trials, China National Knowledge Infrastructure, Chinese Biomedical Literature Database, Chinese Scientific Journal Database and Wan-Fang Database to identify literature of Chinese external treatment for children with FD, and the search period is from inception to July 20, 2022. Besides, we will also search one clinical trial register platforms: ClinicalTrials.gov (www.ClinicalTrials.gov/) for in-progress trials with unpublished data. The search used a combination of subject words and free words, and the search strategy was determined after multiple presearches. The search terms included such as functional dyspepsia, indigestion, children, treatment, intervention and randomization. The detailed search strategy is presented in Table 1.

**Table 1:** Search strategy for PubMed.

No	Search terms
#1	functional dyspepsia [MeSH Terms]
#2	functional dyspepsia [Title/Abstract] OR indigestion [Title/Abstract] OR digestive disorder [Title/Abstract]
#3	#1 Or #2
#4	Traditional Chinese Medicine External Treatment [MeSH Terms]
#5	acupuncture [Title/Abstract] OR moxibustion [Title/Abstract] OR massage [Title/Abstract] OR acupoint application [Title/Abstract]
#6	#4 Or #5
#7	children [MeSH Terms]
#8	children [Title/Abstract] OR pediatric [Title/Abstract]
#9	#7 Or #8
#10	Randomized Controlled Trial [Publication Type]
#11	Randomized [Title/Abstract] OR Randomized Controlled Trial [Title/Abstract] OR Randomly [Title/Abstract]
#12	#10 Or #11
#13	#3 AND #6 AND #9 AND #12

### 2.3. Date collection and management

#### 2.3.1. Selections of studies.

**Figure 1.**
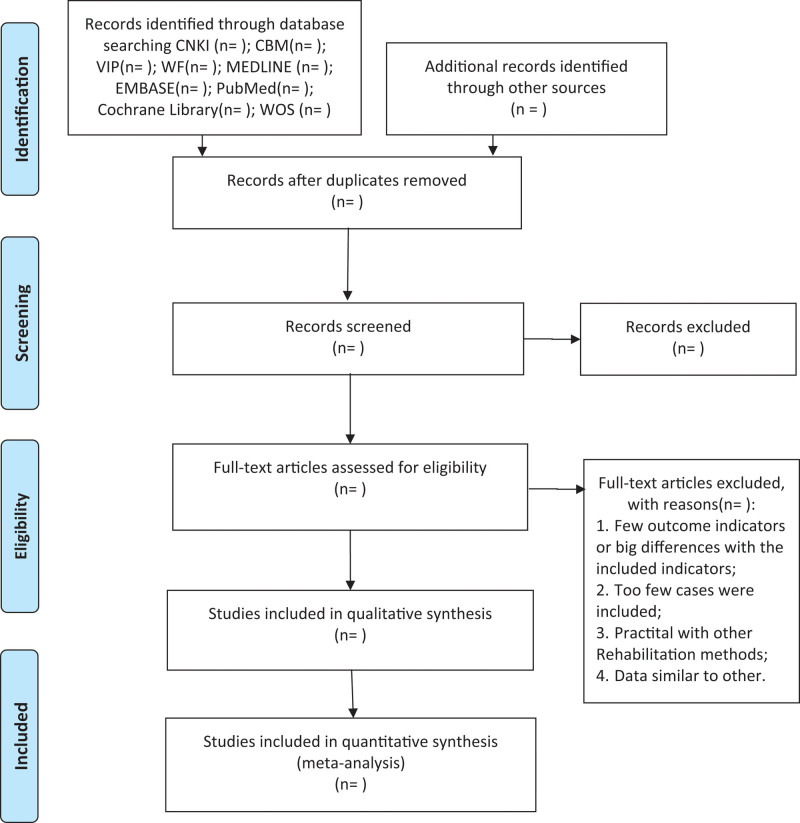
PRISMA flow diagram of study and exclusion. PRISMA = Preferred Reporting Items for Systematic Reviews and Meta analyses.

After completing all the search work, the results will be exported to Endnote software Version X9, and repetitive studies will be deleted by the software. The process of filtering documents will be completed independently by the 2 reviewers and then the eligible literature will be selected separately based on the screening criteria. When differences arise at any stage, we will invite a third reviewer to discuss arbitration. The studies excluded after reading the full text will also be documented and explained why they were excluded. The research flow chart is shown in Figure 1.

#### 2.3.2. Data extraction and management.

Two reviewers will independently select literature and extract data in accordance with the retrieval strategy, and the results will be cross-matched. They will make an Excel to extract literature data, which includes Fundamental information (research title, first author, sample size, age, year, course of disease, treatment period), intervention information (such as treatment method, course of treatment, comparison group, etc.), key elements of bias risk evaluation and outcome indicators. If the differences encountered cannot be resolved through discussion, a third reviewer will be invited to resolve them.

### 2.4. Assessment of risk of bias in the included studies

Two independent reviewers will assess the risk of bias with Cochrane Risk of Bias Tool according to the Cochrane Handbook 5.1.0 for Systematic Reviews of Interventions.

The 2 reviewers will assess each included studies from 7 dimensions, which consist of the risk of bias of sequence generation, allocation concealment, blinding of participants personnel and outcome assessment, incomplete outcome data, selective outcome reporting and other bias. At last, the assessment results will be divided into 3 levels: low risk, high risk, and uncertain risk. If differences arise during the assessing process, they will be resolved through intragroup discussions, contacting authors to determine details with the third-party arbitrator.

### 2.5. Measures of treatment effect

The enumeration data were expressed as relative risk, the measurement data adopted mean difference, and each effect amount was expressed in a 95% confidence interval.

### 2.6. Dealing with missing data

We will e-mail the corresponding author to obtain the necessary information, which is missing or insufficient. If failed, the analysis will be conducted based on the available studies, and we will review the potential impact of missing information.

### 2.7. Assessment of heterogeneity

The heterogeneity of data will be tested by calculating the value of the *I*^2^ statistic. The study is not considered to have large heterogeneous when the *I*^2^ value is <50%. However, when the *I*^2^ value exceeds 50%, there is significant statistical heterogeneity among the trials, and meta-analysis will not be when the *I*^2^ value exceeds 50%, there is significant statistical performed. At this time, subgroup stratification analysis is needed to explore the possible causes of heterogeneity.^[14]^

### 2.8. Assessment of reporting biases

We will use funnel charts to assess reporting biases. When a sufficient number of included studies (at least 10 trials) are available, we will conduct a test for funnel plot asymmetry using the Egger method.

### 2.9. Data synthesis

The synthesis will be performed by generating a forest plot for meta-regression. If the heterogeneity test indicated that there was no substantial heterogeneity between studies, the Mantel–Haenszel method was fitted to calculate pooled estimates, 95% CIs, and combined *P* values. If substantial heterogeneity is indicated by *I*^2^ 50%, the random effects model will be performed using the DerSimonian and Laird method (DerSimonian 1986) and the rma function. The significance of the *P* value represents the strength of evidence against the null hypothesis of no intervention effect. We will network the translated outcomes within studies and specify the relations among the MD across studies, making different comparisons. In addition, the random effects variance and inconsistency variance were roughly equal, which is considered to be less inconsistent.

### 2.10. Subgroup analysis

The subgroup analysis will be conducted to explore different types of TCM external treatments, treatment time, methodological quality, etc. when heterogeneity is high.

### 2.11. Sensitivity analysis

In order to test the robustness of the main decisions in the review process, we will conduct a sensitivity analysis. The main analysis points include the impact of method quality, sample size, and missing data on the study. The meta-analysis will be reused, and more inferior quality studies will be excluded. The results will be compared and discussed according to the results.^[15]^

### 2.12. Grading the quality of evidence

The quality will be evaluated by using the grading of recommendations assessment, development, and evaluation.^[16,17]^ The assessment results will be divided into 4 levels: very low, low, moderate, and high.

## 3. Discussion

FD is common in children, with as many as 80% of those being evaluated for chronic abdominal pain reporting symptoms of epigastric discomfort, nausea, or fullness.^[18]^ Nowadays, many studies have shown that external treatment of TCM not only has advantages in the treatment of FD in children, but also can reduce the recurrence of the disease. Network Meta-analysis, as a method that can directly and indirectly compare multiple related treatment methods, provides an important basis for clinical treatment.^[19]^ As far as we know, there is no high-quality systematic review or network meta-analysis on the treatment of FD in children with external treatment of TCM. Accordingly, we decided to carry out this study to compare the efficacy and safety of different external treatments of TCM for FD in children. Our study will provide clinicians and guideline makers with available evidence on non-pharmacological interventions. The results of this protocol will be published in the relevant journal as soon as possible, and a quick update will be made when supplements are required.

## Author contributions

JC and JW had the original idea of this work and drafted the protocol. The search strategy was developed by all the authors and will be performed by JC and JW, YW, and CZ independently screen the potential studies, extract data from the included studies, assess the risk of bias and complete the data synthesis. GH will arbitrate in cases of disagreement and ensure the absence of errors. All authors approved the publication of the protocol.

**Conceptualization:** Junyu Cui, Jiaxin Wang.

**Data curation:** Ying Wang, Chuang Zhang.

**Formal analysis:** Junyu Cui.

**Funding acquisition:** Zhihong Wang.

**Investigation:** Junyu Cui.

**Methodology:** Jiaxin Wang, Guanyu Hu.

**Supervision:** Zhihong Wang.

**Validation:** Junyu Cui, Guanyu Hu.

**Writing – original draft:** Junyu Cui, Jiaxin Wang.

**Writing – review & editing:** Junyu Cui, Zhihong Wang.
